# Development and Application of Resistance Strain Force Sensors

**DOI:** 10.3390/s20205826

**Published:** 2020-10-15

**Authors:** Yinming Zhao, Yang Liu, Yongqian Li, Qun Hao

**Affiliations:** 1 School of Optics and Photonics, Beijing Institute of Technology, Beijing 100081, China; zhaoyinming@cimm.com.cn (Y.Z.); qhao@bit.edu.cn (Q.H.); 2 Beijing Changcheng Institute of Metrology & Measurement, Beijing 100095, China; 3 Key Laboratory of Micro/Nano Systems for Aerospace of Ministry of Education, Northwestern Polytechnical University, Xi’an 710072, China; ly201169@mail.nwpu.edu.cn

**Keywords:** resistance strain force sensor, resistance strain gauge, stress transfer, creep effect, carbon nanotubes (CNTs), piezoresistive effect

## Abstract

Resistance strain force sensors have been applied to monitor the strains in various parts and structures for industrial use. Here, we review the working principles, structural forms, and fabrication processes for resistance strain gauges. In particular, we focus on recent developments in resistance stress transfer for resistance strain force sensors and the creep effect due to sustained loads and/or temperature variations. Various error compensation methods to reduce the creep effect are analyzed to develop a metrology standard for resistance strain force sensors. Additionally, the current status of carbon nanotubes (CNTs), silicon carbide (SiC), gallium nitride (GaN), and other wide band gap semiconductors for a wide range of strain sensors are reviewed. The technical requirements and key issues of resistance strain force sensors for future applications are presented.

## 1. Introduction

The resistance strain gauge was invented by Simmons and Ruge in 1938 [1], and it has been nearly 80 years since resistance strain force sensors began to be produced in 1942. Force sensors based on resistance strain gauges have been widely used in the stress tests of various components and structures in the aerospace, transportation and automobile industries, civil engineering, and even the medical field. For example, in civil bridge structures, the great amount of strain acting on the bridge structure over a long time will fatigue the structure and yield a fracture. By monitoring the structural strain distribution within bridges, early failure predictions can be made, and the reliability and service life of the bridge structure can be improved [2,3]. Based on a combination of physical quantity-sensitive elements and resistance strain gauges, the physical quantities such as the load and the moment acting on the structure can be obtained indirectly, and other physical quantities such as strain, displacement, bending moment, torque, and acceleration can be measured directly. A strain sensor based on a resistance strain gauge can measure not only the strain on the surface of the structure but also the strain inside the structure. When the strain gauge is embedded in poured concrete structures, the strain state inside these structures can be monitored in real time through external measuring lines [4].

The structural parameters of a resistance strain force sensor directly affect its measurement characteristics. The structural parameters of the sensitive gates and the manufacturing process determine the static and dynamic measurement errors of a force sensor. Although resistance strain force sensors have been developed for a few decades, to promote their use in engineering applications, research remains ongoing to satisfy the increasing demands of their characteristics. One of these research topics is the dependence of the output characteristics on the transverse effects, the end effect, and the grid wire length of strain gauges. Grid wire spacing influences the measurement error fluctuation. Further, the deflection of the grid wire length and diameter results in matrix strain transfer errors. The creep effects due to sustained loading and/or temperature variations have led to various error compensation methods to help develop a metrology standard for resistance strain force sensors, in particular for dynamic measurement of strain force. To improve the measurement accuracy and long-term stability of the strain gauge force sensor system, the finite element method has been used to optimize the structural parameters of the sensitive gate for reducing matrix strain transfer errors. In this paper, we review the recent progress of the micro–nano structure of resistance-strain-sensitive grids and the strain transfer characteristics of the strain-type force sensor. Further, the technical developments and key problems of resistance strain force sensors for their future application demands are explored.

### 1.1. Resistance-Strain-Sensitive Grids

**Figure 1:**
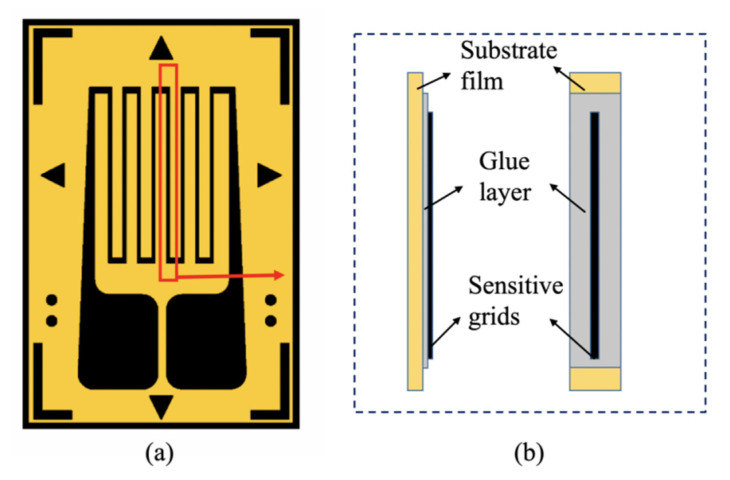
Structure of a metal-foil-type resistance strain gauge: (**a**) top view and (**b**) side view.

Strain-type force sensors are mainly composed of an elastomer, a strain gauge, an adhesive, wire, and a subsequent signal processing circuit. Under application, the resistance strain gauge is adhered to the surface of the metal elastomer [1]. When the elastomer is deformed by an external force, the strain is transferred to the resistance strain gauge through the adhesive. The resistance strain effect or piezo-resistance effect of the resistance strain gauge leads to a change in the resistance values of the sensitive grids. Finally, the transformation circuit transforms the varying resistance into corresponding voltage or current signals [5]. Figure 1 shows the structure of a metal foil resistance strain gauge commonly used in strain-type force sensors. The metal foil is etched into grids and bonded to the polymer substrate film, which is an electrical insulator.

The resistance-sensitive grids of metal-foil-type resistance strain gauges are generally made of constantan, nichrome, and other metal materials, which are the sensitive elements of the stress–strain sensor. The substrate films are made of a polymer material (polyimide, epoxy resin, and phenolic resin) or a glass fiber film, which have good electrical insulation, moisture resistance, and heat resistance. The substrate film supports the sensitive grids and maintains their relative position. During the measurement process, the substrate film transfers the generated strain within the elastomer into the sensitive grid. The coating film protects the sensitive grid from mechanical damage and corrosion due to humidity, which keeps the performance of the strain gauge stable. The coating film, sensitive grids, and substrate film are bonded with a thin layer of adhesive [6,7].

For a rectangular sensitive grid with length *L*, cross-sectional area *A*, and resistivity *ρ*, the resistance is defined as *R* = *ρL/A*. When the action of axial force leads to the wire deformation Δ*L*, the relative resistance change is calculated by:

(1) $$\frac{dR}{R}=\frac{dL}{L}-\frac{dA}{A}+\frac{d\rho }{\rho }$$

According to Poisson’s law, the relationship between the deformation of the cross-section and its length is:

(2) $$\frac{dA}{A}\approx -2\mu \frac{dL}{L}$$

Then, the relative change rate of the radius is:

(3) $$\frac{dR}{R}=(1+2\mu )\frac{dL}{L}+\frac{d\rho }{\rho }$$

The sensitivity coefficient *K* is defined by:

(4) $$K=\frac{dR/R}{dL/L}-1+2\mu +\frac{d\rho }{\rho }/\frac{dL}{L}$$

**Figure 2:**
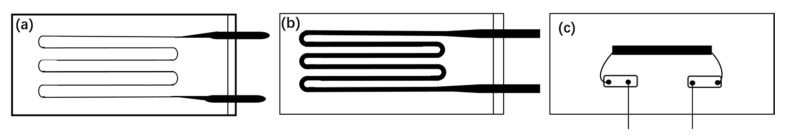
Typical structures of sensitive grid wires in a resistance strain gauge. (**a**) Paper-based wire strain gauge, (**b**) polyimide-based grid strain gauge, and (**c**) semiconductor strain gauge.

The Poisson’s ratio of metal strain materials ranges from 0.3 to 0.5. It has been found that the resistance strain sensitivity coefficient of many alloy materials is greater than (1 + 2µ) [5]. In the early days, for example, for the strain-type sensor invented by Simmons, metal wires were used to create the resistance-strain-sensitive grid, which was placed on the surface of a cylindrical metal elastomer. These strain-type sensors were used to measure the pulse force in a pendulum impact test [1]. However, this kind of wire force strain sensor cannot be used to measure the strain on the surface of the curved parts. As shown in Figure 2a, the sensitive metal wires were adhered to a flexible paper-based material. This flexible strain force sensor can be pasted onto the surface of an elastomer, where the electrical signal passes through two metal wires at both ends of the sensitive grids [8]. The flexible substrate, which has become the basic structure of various resistance strain gauges, enables the sensitive grids to be attached onto the contour surface of an elastomer. The etching process can fabricate certain kinds of grid structures efficiently, in which the controllability of the initial resistance in the grid structures improves the strain gauge sensitivity. As shown in Figure 2b, a foil-like sensing grid was fabricated on an epoxy resin polymer by the etching process. This foil-like grid structure has the advantages of a small creep effect, small lag, and good long-term stability, as well as temperature self-compensation. (1952, Eisler Paul) [5].

Strain gauges made of semiconductor materials have the advantages of a small transverse effect coefficient and small mechanical lag. Their sensitivity coefficient is far greater than that of the metal strain gauge. As shown in Figure 2c, semiconductor strain gauges are often used to make small-scale force-strain sensors.

In 1973, to overcome the inherent shortcomings of normal stress loading sensors, Hogstrom put forward the theory of shear stress sensors. An I-section cantilever was designed to measure the shear stress applied to a loading beam. In 1974, Stein and Erdom established a complete mechanical model to characterize the loading elastomers. According to the calculation results of the mechanical model using the finite element method, the characteristics including strength, stiffness, stress field distribution, and the displacement within elastomers could be analyzed to optimize the loading beam structures.

To develop force sensors that work in a range of several kilograms to dozens of kilograms, an aluminum alloy with a low elastic modulus is used to manufacture elastomers. Furthermore, small range loading sensors were developed using multi beam structures to solve the contradiction between sensitivity and rigidity. Parallel-beam loading sensors are based on the principle of constant moment and utilize the structure of bending stress on the parallel-beam surface, thereby laying a theoretical foundation for the design and calculation of parallel-beam loading sensors.

**Figure 3:**
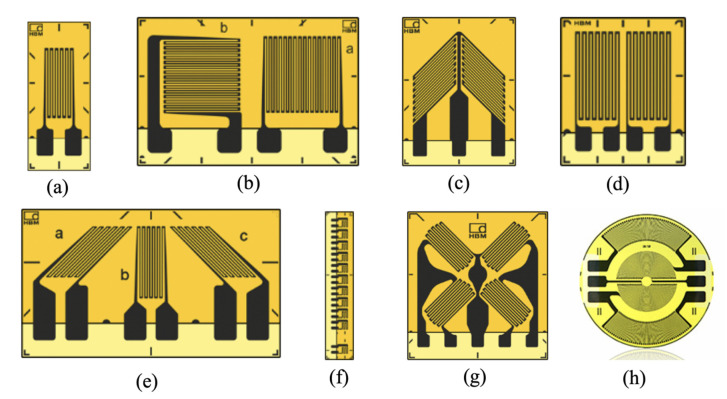
Geometrical structures of various sensitive grids used in resistance strain gauges for different applications. (**a**) Straight strain gauge, (**b**) T strain gauge, (**c**) V strain gauge, (**d**) double-bridge strain gauge, (**e**) triple-grid strain gauge, (**f**) chain strain gauge, (**g**) full-bridge strain gauge, and (**h**) circular-membrane strain gauge.

The original sensitive materials were constantan or nichrome alloy, which were fabricated into wires hundreds of micrometers in diameter. With the progress of the metal rolling process, these sensitive alloy materials can now be processed into foil a few micrometers thick. Compared with wire-sensitive grids, foil-sensitive grid structures can be prepared by the process of photolithography and wet etching into various forms, as shown in Figure 3. Straight grid lines are used for single axis measurements, while T-shaped grid structures are used for biaxial stress or biaxial measurements with known principal stress directions. V-shaped grid structures with two sensitive grids arranged at 90° can measure torsional strain or shear stress. Two bridge resistance strain gauges composed of two groups of sensitive wires arranged parallel to each other are applied to measure curved beams. Three groups of strain-sensitive grids arranged at a fixed angle are used to measure the biaxial stress with an unknown main direction. As shown in Figure 3f, several sensitive grids with equal interval distributions are connected in series to form a chain strain gauge, which is used to measure the strain field with a gradient distribution. Full bridge strain gauges composed of four sensitive grids arranged at 90° to each other are used for biaxial stress measurements of tension and compression or to measure both torsional stress and shear stress in a shear beam. Circular membrane strain gauges composed of sensitive grids along the circumference are mainly used for building pressure sensors [9,10].

The sensitive grid line has a cross-section with a rectangular shape, which is bonded on the polymer substrate. The flexibility of this grid line ensures the strain measurements on surfaces with different curvature radii. The optimized end structures and corner arcs of foil sensitive grids improve their fatigue life and tensile strength and reduce the transverse effect [10].

### 1.2. Materials for Resistance-Strain-Sensitive Grids

The performance indexes for strain-sensitive grid materials are the strain sensitivity coefficient, the resistance temperature coefficient, metallographic structure uniformity, fatigue life, and machinability. These material characteristics of building strain-sensitive grids directly determine the performance of resistance strain gauges.

There are two groups of sensing grid materials: metallic materials and semiconductor materials. The most commonly used semiconductor materials for strain grids include silicon, germanium, antimony steel, gallium phosphide, etc. Metal-sensitive grid materials are copper nickel (Cu-Ni) alloys, nickel chromium (Ni-Cr) alloys, nickel chromium modified alloys, nickel copper alloys, iron base alloys, platinum base alloys, gold base alloys, and handle base alloys. Among these alloys, the Cu-Ni alloy and Ni-Cr alloy have been the most commonly used in recent decades for building variable strain-sensitive grids. The compounding elements of these alloy materials have hardly changed in recent decades. However, improvements in the manufacturing process have greatly improved the performance parameters of these alloy materials. Copper nickel (Cu-Ni) alloy is mainly made of smelted metal components of copper and Cu, along with a small amount of trace metals. The commercial brands of Cu-Ni alloy and their constituent proportions are as follows: constantan of Ni 40%:Ni 60%, Advance of Ni 43%:Cu 57%, and Cupron of Ni 45%:Cu 55%. The constituent ratio of one improved copper nickel alloy is about Ni 44.2%:Mn 1.5%:Fe 0.5%:Cu 53:8% (Hamilton precision metals, Inc. HPM). The resistance temperature coefficient (RTC) of the Cu-Ni alloy is adjusted by the proportion of the constituents and the heat treatment process, which helps prepare resistance strain gauges to self-compensate for the temperature to reduce the variable temperature fluctuations. The effect of the strain sensitivity coefficient’s increase with the temperature can be further compensated for by an external circuit [11]. The sensitivity coefficient of the annealed Cu-Ni alloy remains constant, allowing one to prepare resistance strain gauges with a large measurement range. The material composition of the Ni-Cr alloy is Ni 80%:Cr 20%, and its resistivity ranges from 85 Ohm/cm to 110 Ohm/cm. The resistivity of the Ni-Cr alloy is about twice than that of the Cu-Ni alloy. This larger resistivity value makes the Ni-Cr alloy a suitable material for the small volume chips of resistance strain gauges.

The strain sensitivity coefficient of Ni-Cr alloy maintains values of 2.0~2.1 under the tested temperature environment. When the Ni-Cr alloy gauges work with Platinum wires in self-compensation circuits, their working temperature reaches up to 800 °C. The resistance temperature coefficient of the Ni-Cr alloy is larger than that of the Cu-Ni alloy at room temperature. The former is often used to make temperature gauges for industrial applications, whereas the Cu-Ni alloy used to make resistance strain gauges for normal temperature environments.

Supplementation with small quantities of other metal elements can greatly improve the properties of nichrome alloy. For this purpose, trace elements, such as aluminum, iron, and copper, have been added into nichrome alloys. A nichrome iron alloy, nichrome aluminum iron alloy, nichrome manganese silicon alloy, and other special nichrome alloys have been successfully developed to satisfy specific requirements for practical applications. Among them, the Ni-Cr-Al-Fe alloy (also known as the karma alloy) is adopted as the main sensitive material for high-precision resistance strain gauges. Their resistivity of more than 130 Ohm/cm makes this type of alloy suitable for producing very small strain gauges for applications that require a great resistance value. Their resistance temperature coefficients (RTCs) are approximately linearly proportional to their temperatures. Their RTCs become smaller with the addition of an increasing amount of aluminum [12]. Thus, the karma alloy is suitable for building temperature self-compensating strain gauges [11]. Furthermore, alloy compositions and their process parameters for heat treatment can modulate the strain sensitivity coefficient of the karma alloy. The addition of other metal elements, such as an appropriate amount of aluminum and copper elements, can also improve the processing performance and electrical contact performance of the nichrome (Ni-Cr) alloy. For example, the addition of 1.5% manganese and 1.19% silicon element into the Ni-Cr alloy would improve the alloy’s resistivity up to 140 Ohm/cm.

The strain-sensitivity coefficient of the aforementioned metal alloys ranges from 2.0 to 2.6. The optimization of the metal constituents, the smelting process, and the heat treatment process can enhance the performance of an alloy. The materials composition of Pt-W alloy was optimized to be Pt 92%: W 8% with a greater sensitivity coefficient greater than 5.0 which is suitable for the preparation of high-strain-sensitivity coefficient gauges [13]. An investigation of new stress–strain-sensitive materials would be of great significance for developing stress–strain gauges. The exploration of new sensitive materials and the improvement of existing materials will continue alongside the increasing demands for stress–strain gauges.

### 1.3. Fabrication Technologies for Resistance Strain Gauges

**Figure 4:**
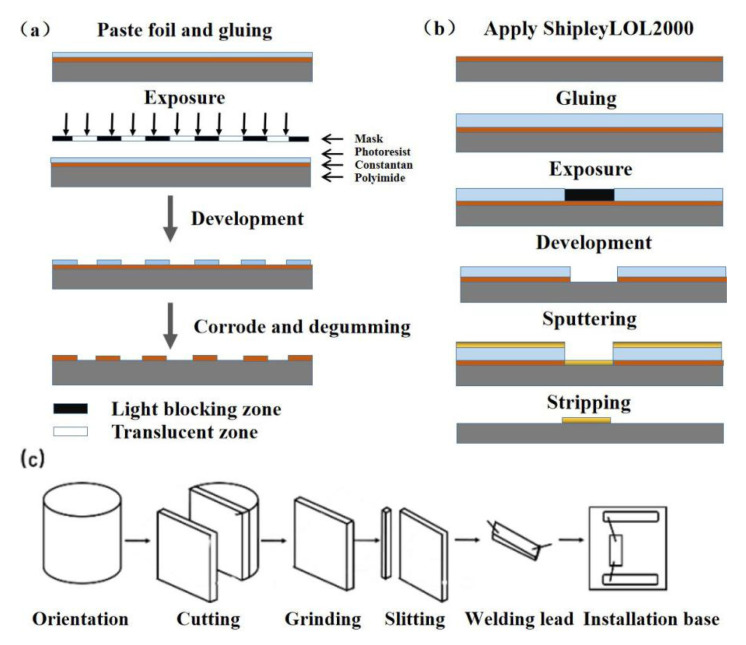
Strain gauge manufacturing process. (**a**) photolithography process, (**b**) sputtering and stripping process, and (**c**) manufacturing process for shaping-type strain gauges.

Wire resistance strain gauges are mainly wound by the multilayer coiling of metal wires. Because of their poor stability, large creep, and large hysteresis effect, wire resistance strain gauges have been replaced by foil strain gauges [14]. Metal foil strain gauges are fabricated by processes such as photolithography, wet etching, and dry etching [15]. Today, most sensitive elements of force strain sensors are made of metal foil strain gauges because of their low creep hysteresis and high stability. The fabrication processes for metal foil strain gauges are shown in Figure 4a and include pasting the foil, gluing, photoetching, development, corrosion, and degumming. Another type of strain-sensitive gauge is a metal film strain gauge, which is made by vacuum deposition and sputtering processes [16]. These processes produce a greater bond strength between the sensitive film material and the substrate, which largely reduce the creep hysteresis in metal foil strain gauges. However, metal film strain gauges are not suitable for mass production due to the need to use expensive processing equipment. Figure 4b shows the main manufacturing steps for metal film strain gauges, including gluing, exposure, development, sputtering, stripping, and degumming.

Semiconductor materials for strain gauges can be of a shaping-type (P-type, N-type, and PN-type), diffusion type, or film type. Semiconductor strain gauges are made of silicon, germanium, antimony steel, gallium phosphide, and other semiconductor materials. Shaping-type strain gauges, for example, are made of monocrystalline silicon material through fabrication processes based on crystals, such as cutting, polishing, and grinding, as shown in Figure 4c, which mainly includes orientation, cutting, grinding, slitting, welding leads, installation of the base, etc. [17].

To fundamentally reduce the hysteresis and creep effect caused by the adhesive layer, thick film resistance strain gauges with a piezoresistance effect are composed of metal and nonmetal oxide materials. These piezoresistant materials are printed on metal or non-metal substrates via a printing and sintering process. In this way, the strain gauges and the elastomer-sensitive elements are firmly bound together. Further, zero temperature drift compensation can be integrated into one chip. These characteristics of integration enable thick film resistance strain gauges to work under high-temperature conditions and in corrosive environments. The ability of these gauges to be mass-produced at a low cost has promoted their wide application in the measurement of force (weighing), pressure, and gas quantity [18,19].

## 2. Stress Transfer Model of Strain Sensors

**Figure 5:**
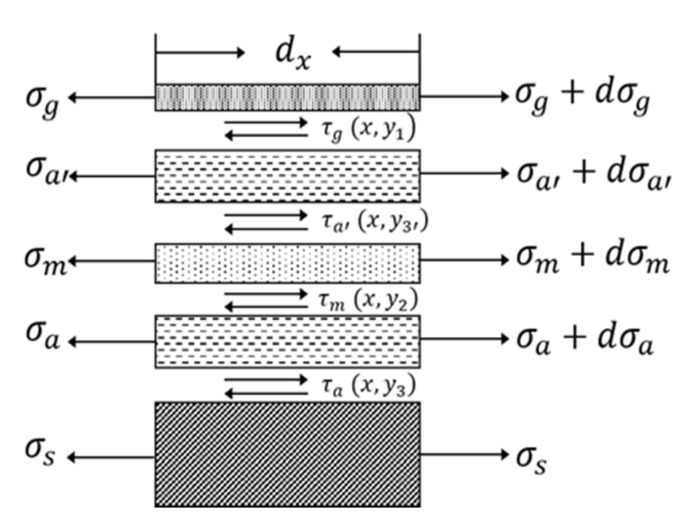
Strain transfer analysis diagram of a resistance strain-type transducer, in which layers of the resistance strain-sensitive grids and two layers of glue are considered. The substrate film and the sensitive grids are bond by the ground adhesive glue and upper adhesive glue in the y-axis direction. The strain propagates along the x-axis direction. The strain transfer characteristics can be analyzed by the elastic-mechanical shear lag theory [22].

The strain transfer model of metal resistance strain-type force sensors considering the double- sided actions of the adhesive layers between the elastomer elements and polymer substrate and between the polymer substrate and sensitive grids is shown in Figure 5. In these structures, the substrate layer and two adhesive layers between the elastomer elements and the resistance strain-sensitive grids are polymer materials. Because of the hysteresis and creep effect of polymer films, there is a certain amount of stress loss during the strain transfer process from the elastomer-sensitive elements to the strain-sensitive grids [20,21]. This stress loss leads to measurement errors between the output value of the strain gauge and the true strain in the elastomer structure. Based on shear lag theory, the analysis model of the strain transfer process can calculate the strain distribution within both the strain gauge substrate and the shear distribution in the adhesive layer. The strain transfer rate can also characterize the effect of the strain transfer [22]. The results show that the average strain transfer rate is determined by the dimensional sizes of the transverse width, the thickness of adhesive layers, the strain transfer transition area at both ends of the sensitive gate, and the elastic modulus of the bonding adhesive layers [23].

The smaller cross-section of the strain grids than that of the elastomer leads to a small perturbation in the strain field distributions on the surface of the elastomer structure’s sticking area. However, when the cross-section of the elastomer elements approaches that of the strain grids, the disturbance of the strain fields within the elastomer cannot be ignored. This indicates that the stress enhancement effect depends on the relative geometric sizes and material properties of the strain grids and the elastomer [24,25].

Based on shear lag theory [26], the shear distribution in the adhesive layer shows that the interactions between the strain grids and elastomer result in deviations from the true value [6,27]. Both the geometric and physical parameters of the adhesive layer and the substrate materials of the strain sensor give rise to a variable transmission rate [27].

**Figure 6:**
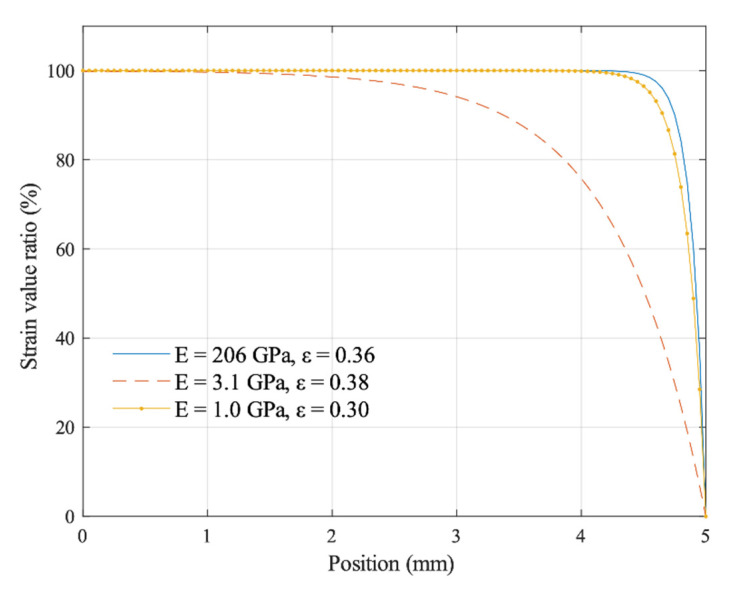
Strain distribution at different positions along the thickness direction. The strain value ratio is obtained from the strain inside the strain gauge and the elastomer.

The concept of strain transfer comes from the research field of composite materials. For composite structures composed of different material layers or materials with different properties, under an external load, the mechanical properties of the strain transfer between the layers of different materials are also different due to their different components. There are many methods to study the strain transfer model, including classical mechanical solutions, finite element simulations, and experimental methods. These methods are used to determine the sensitivity coefficients of strain gauges and assess the errors caused by stress enhancement effects [28]. A strain transfer model for studying the adhesive layers between the elastomer and strain grids is shown in Figure 5. Both the thickness and the mechanical properties of the adhesive layer affect the stress transfer process of the sensor [29]. Figure 6 shows that the elastic modulus of both the steel elastomer and epoxy polymer affects the strain transfer coefficient, which modulates the sensitivity coefficient of the strain gauges according to the differences in the elastomer elastic modulus.

**Figure 7:**
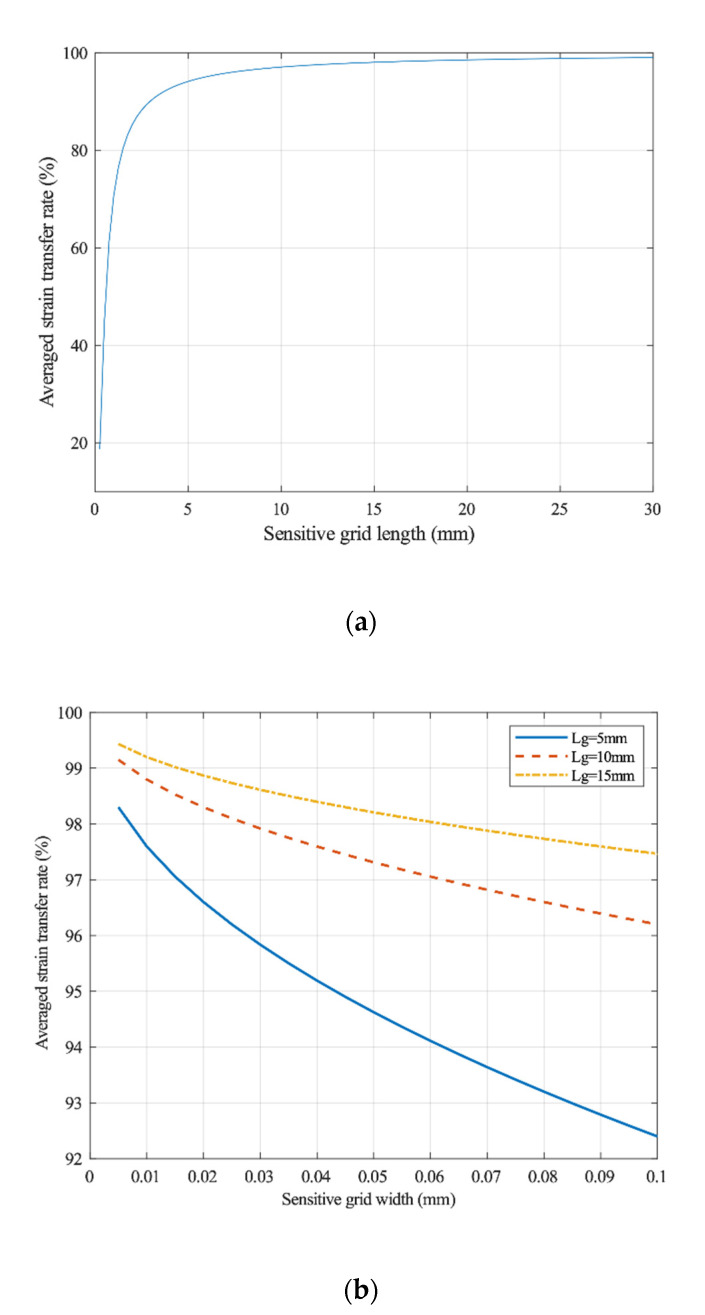
Dependence of the structural parameters of sensitive grids on the variable transfer ratio. (**a**) Sensitive grid length and (**b**) sensitive grid width [22]. *L*_g_ is the length of the sensitive gate.

The numerical analysis method has been used to calculate the dependence of the sensitive grid size on the average strain transfer rate. As shown in Figure 7, when the length of the sensitive grid is less than 5.0 mm, the average strain rate increases sharply. When the length of the sensitive grid exceeds 5.0 mm, the average strain rate approaches the ideal unit one value. A reduction in the width of strain-sensitive grids is beneficial to increase the strain transfer rate [22].

**Figure 8:**
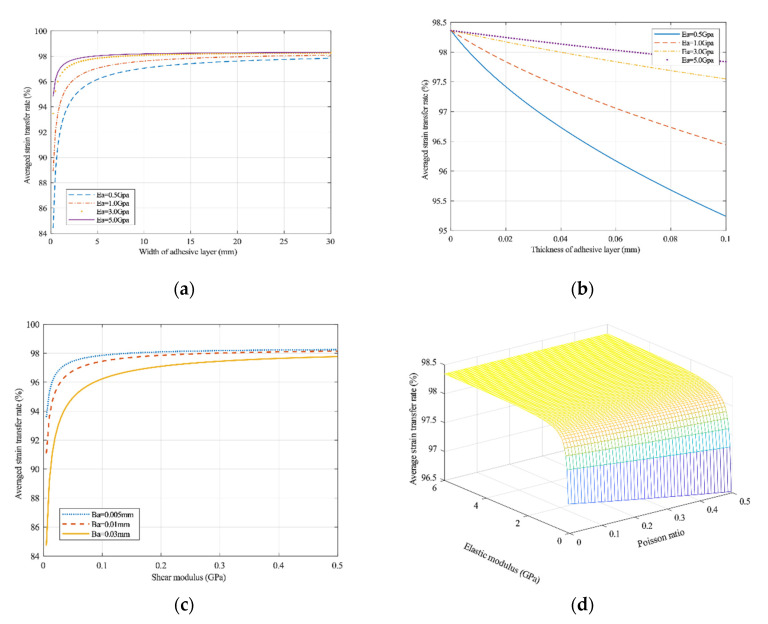
Influence of the adhesive layer parameters on the variable transfer coefficient: (**a**) width of the adhesive layer, (**b**) thickness of the adhesive layer, (**c**) shear modulus of the adhesive layer, and (**d**) bond ratio [22].

As shown in Figure 8, increasing the adhesive layer width can improve the strain transfer rate, whereas the adhesive thickness can reduce the strain transfer rate. However, an increase in the elastic modulus of differently sized adhesive layers can effectively improve the average strain transfer rate. Even when the thickness of the adhesive layer approaches zero, the average strain transfer rate cannot reach that of unit one because the average strain transfer rate is also related to the properties of the substrate material. Enhancing the shear modulus of the adhesive layers will increase the average strain transfer rate under the condition of different adhesive layer thicknesses. Figure 8d shows that Poisson’s ratio for the adhesive layers also determines the average strain transfer efficiency, although Poisson’s ratio has little influence on the average strain transfer efficiency. The elastic modulus of the adhesive layer dominates over Poisson’s ratio of adhesive materials [22].

In the above strain transfer process, the stress reinforcement effect will lead to a strain transition zone at the connecting ends of the adjacent layers, which will reduce the strain transfer coefficient. Compared to surface-bonded strain gauges, the present technique is a better method for embedding strain gauges into elastomers to reduce the stress reinforcement effect. In the experiments on embedding strain gauges into an elastomer with a different elastic modulus, it was found that increasing the elastic modulus of the strain gauge and cementation layer can reduce both the local stress concentration and the transition zone [30,31]. However, the sensitivity of the embedded strain gauge becomes lower than that of the strain gauges bonded on the surface of the elastomer.

When introducing the size ratio parameter between the strain gauges and sensitive grids, the strain transfer formula indicates that the measurement errors caused by strain reinforcement effects becomes predictable [32]. When the strain gauges maintain constant sizes, increasing the elastic modulus of the elastomer helps to reduce both the stress enhancement effect and the length of the strain transition region. When the thickness of the strain gauges and the length ratio of the strain gauges and sensitive grids are constant, increasing the size of the strain gauges will also reduce the stress enhancement effect [32].

## 3. Creep Effects and Error Compensation of Strain Force Sensors

“Creep effects” refer to the output changes during the testing progress when the resistance strain force sensor bears a constant mechanical strain while other external environmental factors remain constant. The deviations due to creep effects reduce the long-term stability of strain force sensors. In the strain transfer model of a resistance strain force sensor shown in Figure 5, the adhesive polymer materials dominate the contributions to the creep effects due to their relaxation characteristics under long-term loading or a high-temperature burden. Creep effects are also affected by the structures of the elastomer and strain grid configurations along with their preparation process and the bonding process of the adhesive layers. During strain measurements under high-temperature conditions, especially under the conditions of long-term monitoring, the measurement errors resulting from creep effects must be considered [33]. A creep compensation resistance strain gauge was developed by optimizing the geometric dimensions of the sensitive grids of the resistance strain gauge. This is a common method to optimize the sizes of sensitive grids to reduce creep effects in small-range resistance sensors by analyzing a one-dimensional mechanical model and optimizing the strain transfer coefficient [33]. Error compensation for creep effect reduction can be realized by combining the methods of curve fitting and piecewise linear interpolation [34].

Creep effects produce comprehensive errors resulting from the material characteristics of sensitive grids, elastomer elements, adhesive layers, and their geometric and loading conditions [35]. Due to the nonlinear output mechanisms resulting from these creep effects, a neural network has been used to compensate for creep errors because of its self-learning and nonlinear compensation characteristics [36]. By training the compensation parameters of neural network models, creep errors can be partially compensated for, and the properties of long-term stability can be improved [37]. To engage in real-time error compensation for the creep effect in a strain force sensor, a radial basis function (RBF) neural network model was established to compensate for creep effect errors [38]. The experimental results show that an RBF neural network can reduce creep effect errors to less than 0.005% in force sensors. However, to obtain an ideal compensation effect, a large number of initial data samples and much training progress are needed to obtain the parameters of the neural network models, which are too complex and unreliable in their initial stages [39]. The long-term creep effects of polymer composites became predictable when a viscoelastic constitutive model was built from short-term testing data [40]. The linear viscoelastic constitutive model helped to derive a multi-layer interface strain transfer function expression to obtain the strain transfer loss and creep effect errors in optical fiber sensors [41]. Creep recovery experiments using graphite epoxy resin composite materials showed that the prediction results, in theory, are consistent with the experimental data when the loading time is less than 1000 min. However, the differences between the prediction results and the measurement results became obvious (as high as 10%) when the loading time lasted longer than 10,000 min [40].

The key point for performing creep compensation is understanding the creep characteristics of the individual parts of strain sensors, including the elastomer, adhesive layers, substrate layer, and the sensitive grids. The mutual combination of opposite sides can minimize all creeps to an acceptable level. A reverse compensation algorithm was proposed to perform error compensation for the creep effects in strain sensors. When the creep characteristics of strain gauges are opposite to the inherent creep value of the elastomer, negative compensation can reduce creep errors [42]. Because of the complexity of the creep characteristics in strain force sensors, it is difficult to absolutely eliminate such errors using various hardware self-compensation methods. The combination of structure optimization and a corresponding intelligent algorithm was designed to meet the compensation requirements of force sensors. Combined with an open-loop control circuit, a reverse compensation algorithm was introduced to compensate for both the hysteresis and creep transfer characteristics in resistance strain force sensors, which have difficulty minimizing creep effects and lag effects at the same time [43]. This method effectively reduces the linear errors caused by lag effects and creep effects [44]. The Kelvin Voigt model describes the energy interactions between the physical effects and structures of force sensors. This model reveals the dependence of the initial loading strain on hysteresis and creep effects, from which the corresponding compensation algorithm can be derived [45].

The creep characteristics in metal strain force sensors involve complex mechanisms and serious nonlinear errors, which are related to the structure, shape, preparation processes of the elastomer and sensing elements, the performance of the adhesive and its curving temperature, and even the loading and environmental conditions. High-temperature stress–strain materials that are likely to effectively eliminate these creep effects are currently in development.

## 4. CNTs-Based Strain Sensors

New materials for novel strain-sensitive mechanisms need to be explored due to the growing number of applications for this technology. Silver nanoparticles and carbon nanotubes are in development for compressible flexible piezoresistive sensors [46,47]. Especially, the carbon nanotubes (CNTs) have demonstrated utmost interest for sensing applications due to their large specific surface area [48,49]. The exceptional sensitivity to their environment medium or varying conditions, the CNTs deposited on rigid substrate films have been demonstrated to be various mechanical sensors [50], and chemical or biological sensors for analytical species [51].

Carbon nanotube composite films are usually fabricated by mixing small amounts of single-walled or multiwalled carbon nanotubes with selected polymers [48]. The flexible CNTs sensors have the significant mechanical robustness, including high Young’s modulus and high tensile strength [49,50,51,52]. These promising electromechanical properties enable carbon nanotube devices to provide long-lasting, reliable devices compatible with industrial applications.

However, their structure complexity of CNTs-based nanodevice fabrication results in the variability of their mechanical properties or electrical properties. To overcome the hinders on the progress of nanodevice fabrication, significant efforts have been expended on the production of CNTs macrostructures [53]. The long multi-walled CNTs ropes are prepared using the floating catalyst chemical vapour deposition fabrication method. The resulting mechanical properties reach an average tensile strength of 210 MPa and Young’s modulus of 2.2 GPa, respectively [50]. Even exposed to environment condition of the temperature at 450 °C, the tensile strength up to 300 MPa has been maintained for an hour [54]. This temperature stability of the multi-walled carbon nanotube is attributed to its long yarns structures with different twisted and interwoven patterns [54].

The prediction in theory indicates that a single-walled carbon nanotube deposited on either rigid or flexible substrates possesses extremely high tensile strengths. The estimated strain at tensile failure of a single-walled carbon nanotube reaches up to as high as about 30% [52], and thus resulting in an expected tensile strength of 300 GPa. The measured tensile strengths of individual multi-walled carbon nanotube macrostructures have been conducted, which expect that the Young’s modulus of single-walled carbon nanotube varied from 270 to 950 GPa [52]. The dimensional dispersion and waviness of the CNTs array contribute to the poor modulus of composited materials. The individual CNTs of several microns have been found to translated its outstanding mechanical properties into the CNTs reinforced composites [55]. Along with the advance in CNTs growth techniques, the tensile properties of millimeter-long MWCNTs were directly measured. The average tensile strength and Young’s modulus of the CNTs were measured to be 0.85 GPa and 34.65 GPa, respectively [56]. The CNTs devices exhibit promising values of tensile properties for building a high modulus/strength composite material, which promotes their applications to be used as strain reinforcement resistance strain sensors.

Along with the advance in flexible electronics, the CNTs-based sensors provide robust strain force sensor networks for infrastructure health monitoring [57,58] and environmental monitoring [59]. There are various flexible CNTs sensors proposed for the next generation of wearable devices for human welfare monitoring [60,61].

Owing to the interesting mechanical and electrical properties of the carbon nanotubes, various strain gauges [62,63] and chemiresistor sensors [64] have been yielded. However, the challenge for industrial requirements of CNTs-based flexible sensors lies in their device uniformity or reproducibility. The reproducibility addresses the standard deviation both in initial device resistance, in the fabrication process, and in device sensitivity [65]. Most of sensors reported to this day, either for mechanical or chemical sensitivity, are fabricated by inkjet printing processes on flexible substrates, such as polydimethylsiloxane (PDMS), polyethylene terephthalate (PET), and other polymer materials. Although the gauge factors range from a few tens to hundreds for the flexible strain films, the stability on the fabrication process and the variability in the device sensitivity are the issues of the CNTs sensors. A few strain sensors deposited on glass substrates possess the resistances range and the repeatability over loading cycles [63,66]. No satisfying analysis of stability on device properties is attributed to the variability of film quality [66,67]. The in-situ characterization of multi-walled carbon nanotube under external strain has uncovered the prominent mechanisms of CNTs motions [62]. Inkjet printing techniques have provided outstanding results for flexible resistance sensors in terms of their resistance reproducibility [68]. The carbon nanotube networks (CNNs) fabricated by inkjet-printing present potential applications for flexible resistance strain sensors [69]. With the advance in the fabrication process of CNTs-based films, the CNTs based flexible strain sensors have a huge demand in medical health instruments and wearable devices.

## 5. Piezoresistive Effect in Wide Band Gap Semiconductors

Metal sensitivity material have been commercialized and are widely employed for the strain gauges. The piezoresistive effect in metals fundamentally depends on the geometry deformation under the external applied strain. The typical gauge factors of metallic mechanical gauges are less than tens when subjected to mechanical deformations. In contrast to the metal sensitivity materials, the wide band gap materials, e.g., silicon carbide (SiC) and gallium nitride (GaN), have both piezoelectric and piezoresistive properties [70,71]. The carrier mobility in these materials dominates the piezoresistive performance, whereas the geometry deformations are neglectable. Furthermore, these materials have inertness to corrosive chemicals and high temperature due to the property of wide band gap.

Both the hardness properties and thermal conductivity from all forms of SiC are better than that of the metal sensing materials. The Young’s modulus of SiC materials reaches up to typical values of 450 GPa [71]. The superior properties in SiC material are derived from the high strength of the Si–C bond. There are a large number of polytypes crystal structures built from the Si-C bonding unit. Of these, the most attractive devices are the 4H, 3C, and 6H polytype due to their excellent material properties. Compared with the band gap of 1.12 eV for silicon, the band gap of 4H SiC increases threefold up to 3.23 eV at room temperature [71]. The wide bandgap value allows SiC based devices remain stability at high temperature because few numbers of free electrons are formed from thermal activation. The 4H SiC-based MEMs sensors are well demonstrated at temperature up to 500 °C in the sensing of motion, acceleration and gas flow [70,72]. The 3C SiC polytype is commonly used in MEMs-based sensors due to its efficient growing on Si wafers [73,74]. The excellent electronic and mechanical properties of SiC materials enable these materials many possibilities for a wide range of harsh environment sensors. These hostile applications featuring high temperatures includes the control operations of internal combustion engines, compressors and turbines.

The large piezoresponse, combined piezoelectric effect and piezoresistive effect, have been utilized as mechanical sensing technologies, where the effects depend on the carrier mobility in the wide band gap materials [75]. For the ultrasensitive sensing technology, the enhancement of the piezoresistive effect and/or the piezoelectric effect are crucial important. The optimal crystal orientations or the doping concentrations in materials have been of great interest for improving their piezoresistive sensitivity [70,76]. For example, the gauge factor of single crystalline 3C-SiC is 5.0 and 30.3 in the [100] and [110] orientation, respectively [77,78]. For a GaN-based electromechanical devices, the time-dependent piezoresponse is much larger than that of other semiconductors because of the large piezoelectric contribution to the overall piezoresponse response. However, the static piezoresistive effect of a GaN-based device is small. The gauge factor of GaN-based piezoresponse strain gauges is 167 at room temperature and remains 104 at 250 °C [75]. Hence, GaN-based sensors with a large gauge factor has an advantage over other piezoresistive materials for time-dependent dynamic applications [79].

Interesting, the coupling effects among the physical effects, such as coupling piezoresistivity with piezoelectricity, have emerged to be a promising approach to boot the sensitivity effect. A giant piezoresistive effect resulting from the optoelectronic coupling in a cubic silicon carbide/silicon heterojunction gives rise to a gauge factor of 58,000, which is more than 2000 times greater than that of cubic silicon carbide [80]. The coupling effects between the diverse physical effects maybe promote the development for the ultrasensitive sensing technology.

Recent progress in nanomaterials and nanotechnologies has indicated that nanowires and nanoparticles may become the basic building blocks for nanoscale electronic systems. When the dimensional sizes of piezoresistive sensors are reduced down to the nanoscale level, the increasing of both the charge mobility and surface-to-volume ratio would bring about a meaningful enhancement of the piezoresistive effect [81,82]. For instance, the piezoresistive coefficient of *p*-type silicon nanowires sharply increases up to −3550 × 10^−11^ Pa^−1^ in comparison with value of −94 × 10^−11^ Pa^−1^ in a bulk silicon [82,83]. The giant piezoresistive effect has been observed in silicon nanowires [83,84] and ZnO nanowires [72]. With their excellent characteristics of flexibility and large piezoresistive sensitivity, the nanocomposite materials show promising applications for high sensitivity flexible tactile sensors [84]. The precise constituents of the nanomaterials determine their electrical and mechanical properties. By sufficiently finely tuned nanowires in uniform phase-change materials thin films, its significant piezoresistive effect gives rise to a giant gauge factor of 338 used in integrated flexible tactile sensors [85]. The typical applications of these tactile sensors are electronic skin, health monitoring, and energy harvesting.

## 6. Conclusions and Prospects

The strain transfer and creep characteristics of strain sensors are the key factors that determine the sensitivity coefficient, mechanical lag, and long-term stability of strain sensors. This paper mainly introduced the recent research progress on this key technology, which will contribute to related research topics and the development progress of strain sensors.

The materials for preparing strain-sensitive grids are one of the key topics for developing high-precision strain sensors. Future work should be carried out in such fields as the ingredient technologies for sensitive grid materials, forging manufacturing technology, heat treatment and mechanical stability technology, and surface treatment technology for improving the characteristics of sensitive grid materials.

The properties of resistance strain gauges should be large resistance and low power consumption to develop high-precision resistance strain gauges. New sensitive grids for large sensitivity values with the function of temperature compensation should be made of barium and niobium rather than materials made only from nickel. Various error compensation technologies and software algorithms could reduce creep effects to ensure the high-temperature stability of resistance strain gauges. Resistance strain force sensors are likely to be used innovatively for the measurement of dynamic impact force, especially in the field of high-speed rail crash testing, where the sensitive properties of gauges, creep effects, and the transverse effect should be comprehensively analyzed. Due to the increasing applications of strain force sensors in high-temperature, high-pressure, ultra-low temperature, and other intolerable environments, it is necessary to develop special strain force sensors that can work under such harsh environments. Micro-electro-mechanical system (MEMS) strain sensors have the advantages of being resistant to harsh working environments and have been applied to aerospace bridge construction [86,87,88,89]. A high-performance MEMS force sensor was also applied to measure the flow fields behind an aircraft engine [90]. The key factors are the stress–strain characteristics of thin-film sensitive materials and the fast responses of these sensitive elements on a curved substrate.

The main stability and reliability indexes of strain force sensors are determined by the manufacturing technology and manufacturing process, which should be the foci for new strain sensor technologies. In terms of the reliability and packaging technology of strain sensors, the mutual coupling of mechanical, thermal, and electromechanical stress among the packaging structures are a recent problem for developing strain sensors with microscale dimensional sizes. The typical failure types and corresponding failure analysis methods for microscale sensors are thus becoming urgent topics. The test methods for, and reliability evaluation of, stress–strain sensors at the microscale and nanoscale are particularly urgent.

A challenge to provide reliable devices compatible with industrial applications lies in their device compatibility and uniformity on fabrication processes, especially for novel strain-sensitive mechanisms materials, including the CNTs materials and various wide bandgap semiconductors. The carbon nanotube devices fabricated by inkjet-printing present potential applications for flexible resistance strain sensors. These flexible strain sensors have a huge demand in medical health instruments and wearable devices.
